# Comparison of the Penetration Depth of 905 nm and 1064 nm Laser Light in Surface Layers of Biological Tissue Ex Vivo

**DOI:** 10.3390/biomedicines11051355

**Published:** 2023-05-04

**Authors:** Leon Kaub, Christoph Schmitz

**Affiliations:** Department of Anatomy II, Faculty of Medicine, Ludwig-Maximilians-University Munich, 80336 Munich, Germany; leon.kaub@med.uni-muenchen.de

**Keywords:** laser therapy, musculoskeletal system, 1064 nm NIR laser, 905 nm NIR laser, continuous wave lasers, pulsed lasers, porcine/bovine tissues, tissue penetration depth

## Abstract

The choice of parameters for laser beams used in the treatment of musculoskeletal diseases is of great importance. First, to reach high penetration depths into biological tissue and, secondly, to achieve the required effects on a molecular level. The penetration depth depends on the wavelength since there are multiple light-absorbing and scattering molecules in tissue with different absorption spectra. The present study is the first comparing the penetration depth of 1064 nm laser light with light of a smaller wavelength (905 nm) using high-fidelity laser measurement technology. Penetration depths in two types of tissue ex vivo (porcine skin and bovine muscle) were investigated. The transmittance of 1064 nm light through both tissue types was consistently higher than of 905 nm light. The largest differences (up to 5.9%) were seen in the upper 10 mm of tissue, while the difference vanished with increasing tissue thickness. Overall, the differences in penetration depth were comparably small. These results may be of relevance in the selection of a certain wavelength in the treatment of musculoskeletal diseases with laser therapy.

## 1. Introduction

There is increasing evidence that using near-infrared (NIR) laser light to treat patients suffering from musculoskeletal diseases can be beneficial [1,2]. It has been hypothesized that NIR laser light can reduce pain, decrease inflammation and stimulate tissue healing, which is favorable in the treatment of various musculoskeletal diseases [1,2,3,4,5]. NIR light is used in laser therapy because the NIR wavelengths coincide with the so-called therapeutic window, in which light absorption in biological tissue is comparably low and the penetration depth therefore accordingly large [6]. However, there are also differences in the penetration depth within this therapeutic window that depend on the exact absorption and scattering spectra of the tissue [7].

One direct possibility to investigate the penetration depth in biological tissue is to measure the transmission of a laser light source through tissue specimens of varying thickness (e.g., [8,9,10]). In a previous study [8], the penetration of NIR laser light from two laser therapy devices (LTDs) into several different animal tissues was compared. The two LTDs were operated with the same wavelength (905 nm), but had differences in other beam parameters such as pulse length, peak power and beam profiles. The differences in these parameters had only little effect on the penetration of the average light power [8]. One of the LTDs investigated in this previous study was capable to emit laser beams with two additional, different wavelengths (800 nm and 970 nm). A comparison of the different wavelengths showed that 800 nm and 905 nm light reached similar penetration depths, while 970 nm light came significantly less deep. This was attributed to a peak in the absorption spectrum of water at 976 nm [8].

Other commercially available LTDs offer laser light with a wavelength of 1064 nm. Some authors have argued that light with this wavelength has a higher penetration depth than light with a lower wavelength [11]. However, there is a lack of information about the penetration depth of light with a wavelength greater than 1000 nm into biological tissue [12]. Furthermore, one has to keep in mind that light that is used in the treatment of musculoskeletal diseases needs to transmit through the skin barrier, which consists of the epidermis, the dermis and the subcutaneous layer. Depending on the treatment target, the light needs to additionally transmit through some amount of other tissue such as muscle tissue. The light then needs to reach not only the surface layer of the target structure, but also to transmit into the target structure. The main absorbing molecules that need to be overcome in order for the light to reach deep target structures are generally thought to be water, lipids, melanin and hemoglobin [13]. Absorption spectra of these molecules have been investigated in many studies [7]. The absorption of light in lipids is approximately equal for the two wavelengths 905 nm and 1064 nm [7,14]. Since the absorption of light in melanin and hemoglobin decreases with an increasing wavelength, it is often concluded that 1064 nm light can penetrate tissue deeper than light with a shorter wavelength [12]. However, the absorption of water needs to be considered, too. The absorption of water at 1064 nm is approximately two times stronger than at 905 nm [15], which partially compensates the lower absorption of melanin and hemoglobin. Since melanin is only found in the epidermis but water is ubiquitously found in most tissues, it cannot directly be concluded that 1064 nm light penetrates all tissues better than lower wavelengths when comparing these two molecules. In addition, there are large differences in the melanin content of skin tissue between different ethnicities and even between different body parts of an individual [16]. In contrast, hemoglobin is also ubiquitous in tissue, and the lower absorption of light in hemoglobin at higher wavelengths could lead to a difference in the total absorption of tissue. Measurements of the absorption spectrum and penetration depths of 1064 nm light into real tissue are missing.

The present study tested the hypothesis that laser light with a wavelength of 1064 nm penetrates skin tissue better than 905 nm laser light, while there is no difference between these two wavelengths in the ability to penetrate into other tissues. This would be of high relevance for treatments of musculoskeletal disorders with laser light, since any light that is supposed to treat pathologies that are deep underneath the skin needs to be able to overcome the skin barrier.

It should be mentioned that many commercially available LTDs emit laser light that differs in a variety of parameters. Besides different wavelengths, LTDs often offer continuous wave (CW) and/or pulsed wave (PW) laser light. Pulsed lasers allow higher peak powers and can reach into deep layers of tissue with less undesired surface heating than CW lasers [2]. In general, pulsed laser light tends to lead to better clinical results than CW laser light [17]. On the other hand, there are also large differences between the different ways LTDs generate pulsed laser light. Some LTDs emit ultra-short pulses combined with large peak powers, while other LTDs emit very long pulses with peak powers that are only slightly larger than the average power [8]. Two LTDs that both offer pulsed laser light might therefore emit highly different laser beams, which could lead to different and/or less-effective treatments. The LTDs used for the present study were therefore thoroughly characterized before they were employed for the penetration depth measurements.

## 2. Materials and Methods

Penetration depths were measured for porcine skin tissue and bovine muscle tissue following the protocol of an earlier study [8]. The porcine tissue consisted of skin, subcutaneous fat and muscle tissue. The proportions of each layer were approximately equal for all porcine tissue specimens (skin, 3 mm; subcutaneous fat, 6 mm; muscular tissue, remaining thickness). All porcine tissue specimens contained the skin layer. The bovine tissue contained mainly muscle tissue. Since the tissue specimens were freshly acquired from a butcher shop in Munich (Germany), no ethics approval or registration of the present study was necessary. Seven tissue slices, ranging in thickness from 3.3 mm to 21.7 mm (porcine tissue) and from 2.1 mm to 17.0 mm (bovine tissue), were cut from each type of tissue. A medical ultrasound device (HS-3000, Honda Electronics, Toyohashi, Japan) was used to assess the thickness of each specimen before and after the penetration depth measurements. Each thickness measurement resulted from averaging the thicknesses at three positions. For data analysis, the mean of the two thickness measurements (before and after) was computed.

**Figure 1:**
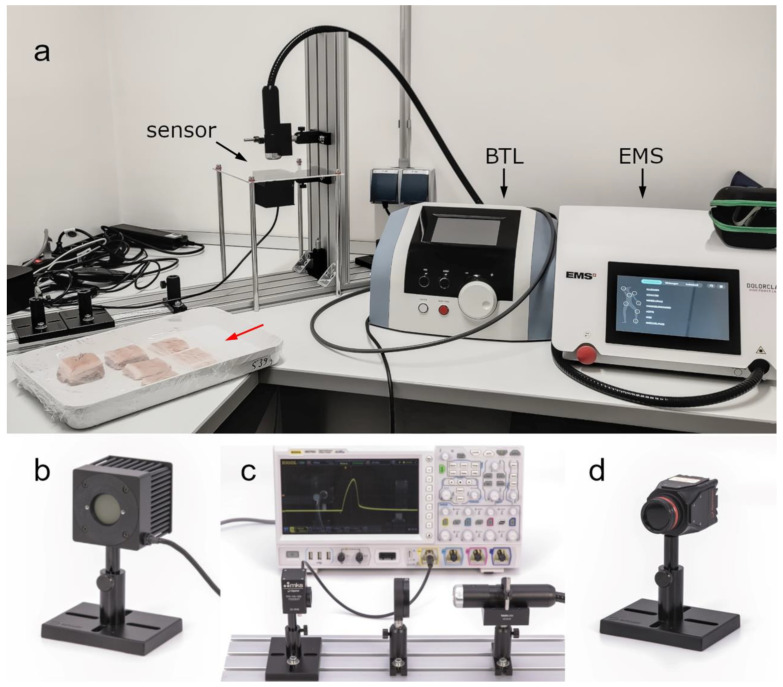
Equipment used in the present study: (**a**) experimental setup of penetration depth measurements showing both the BTL laser (BTL) and the EMS laser (EMS); the thermal power sensor (sensor) in its location for penetration depth measurements as well as the porcine tissue specimens (red arrow) are also shown; (**b**) thermal power sensor; (**c**) photodiode sensor connected to an oscilloscope measuring pulses emitted by the EMS laser; and (**d**) beam profiling camera. Details are in the text. Panels (**b**–**d**) were modified from [8] with permission from the authors.

Two commercially available LTDs were used for the present study: (i) Dolorclast High Power Laser (Electro Medical Systems, Nyon, Switzerland) (hereafter: EMS laser) and (ii) BTL-6000 (BTL, Prague, Czech Republic) (hereafter: BTL laser). Both LTDs can be seen in the experimental setup for penetration depth measurements shown in Figure 1a. Selected specifications of the LTDs provided by their manufacturer are given in Table 1.

The EMS laser was thoroughly investigated in two earlier studies [8,18]. For the characterization of the BTL laser, the same measurements as described in these studies [8,18] were performed in the present study. Three different sensors were used in the present study to characterize the BTL laser (c.f. [18]). Light power was measured using a thermal power sensor (Model 50(150)A-BB-26-PPS; Ophir Spiricon Europe GmbH, Darmstadt, Germany; Figure 1b) that was operated with software from the manufacturer (StarLab 3.62 Build 1; Ophir Spiricon Europe GmbH). Temporal profiles were recorded using a fast photodiode sensor (FPD-VIS300, Ophir Spiricon Europe GmbH) connected to an oscilloscope (MSO7024; Rigol Technologies Inc., Suzhou, China). The temporal profiles were recorded with an optical diffusor (DG20-220-MD, Thorlabs GmbH, Bergkirchen, Germany) in the beam line to protect the photodiode from large power densities (Figure 1c). A beam profiling camera (LT665; Ophir Spiricon Europe GmbH; Figure 1d) was used to record spatial intensity distributions. Software from the manufacturer (BeamGage Professional v6.17.1; Ophir Spiricon Europe GmbH) was used to operate the camera. Beam profiles were extracted from the spatial intensity distributions using the same software.

**Figure 2:**
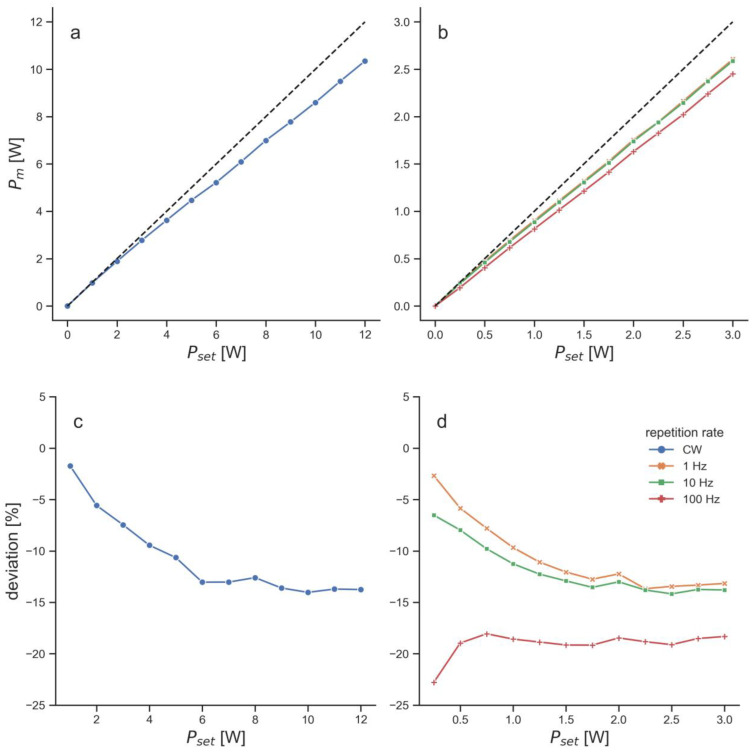
Power measurements of the BTL laser: (**a**,**b**) measured average power ($P_{m}$) for different power values set at the BTL laser ($P_{set}$). Black dashed lines in (**a**,**b**) illustrate ideal curves, in which the measured average power would equal the set power; (**c**,**d**) deviation in percent of $P_{m}$ compared to $P_{set}$. Measurements are shown for continuous-wave (CW) mode (**a**,**c**) and for pulsed-wave (PW) mode at three different repetition rates (**b**,**d**).

For the penetration depth measurements, the light emitter was pointing from above onto the specimen and the thermal power sensor was placed underneath the specimen holder (see Figure 1a). Both LTDs were set to the maximum repetition rate. The EMS laser was set to 40 kHz, which resulted in 1.2 W measured average power. The BTL laser was set to a repetition rate of 100 Hz, a beam size of 1 cm^2^ and a peak power of 6 W, which also resulted in a measured average power of 1.2 W (Figure 2).

The BTL laser beam for the penetration depth measurements was slightly too large to be adequately measured with the thermal power sensor. Unfortunately, it was not possible to adapt the beam size of the BTL laser (addressed below). Therefore, a constant beam size of 20 mm had to be assumed for the empty measurements with this LTD. The transmittance measurements were used to compute penetration depths by fitting a curve according to the Beer–Lambert law using the method of least-squares. Since for the present study the sensor was located outside of the tissue, the correction method from [8] was used to correct for power losses that incurred due to reflections at the tissue—air interface. Penetration depths were then computed for the uncorrected and corrected data.

After measurements with the power sensor, the photodiode was used in the same position to record temporal profiles of the laser beam pulses after transmitting through the specimen. The signals recorded with the photodiode were all smoothed using a moving average with a window length of 100 samples.

All data analysis was carried out with self-written software using the programming language Python (version 3.9, Python Software Foundation, Delaware, MD, USA).

## 3. Results

**Figure 3:**
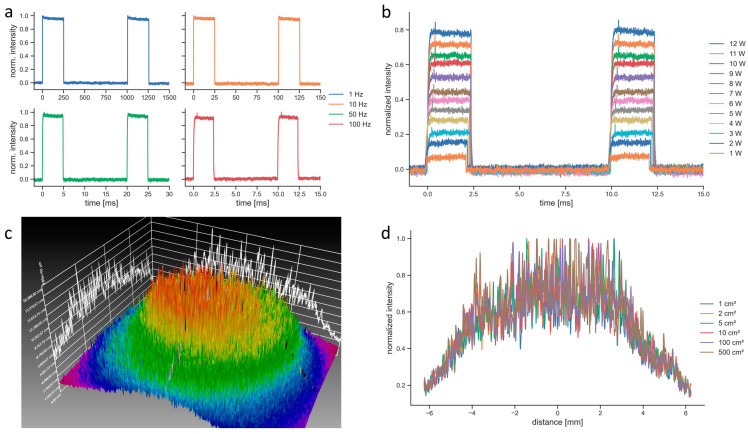
Characterization of the laser beam emitted by the BTL laser: (**a**) temporal profiles at different frequencies and the same peak power (12 W); (**b**) temporal profiles at the same frequency (100 Hz) at different values of peak power; (**c**) spatial intensity distribution; and (**d**) beam profiles along the horizontal line for different set beam sizes (zero indicates the center of the spatial intensity profile shown in (**c**)).

The BTL laser in PW mode was modulated with a rectangular function and emitted light for 25% of the period (duty cycle of 25%), which made the pulse length a function of the repetition rate (Figure 3a). For a repetition rate of 100 Hz, as it was used for the penetration depth measurements, the pulse length was 2.5 ms (Figure 3b). The spatial intensity of the BTL laser followed a Gaussian distribution (Figure 3c). The BTL laser allowed setting the beam size from 1 cm^2^ to 500 cm^2^. However, regardless of the set beam size, the spatial intensity distributions stayed constant as it can be seen in the measurements of horizontal beam profiles (Figure 3d). The camera images were also identical for all tested beam size settings. Therefore, the beam size setting of this device was not functional.

The measured average power of the BTL laser was consistently below the set average power for all set values (Figure 2). At the repetition rate that was used for the penetration depth measurements, the measured power was approximately 19% below the set power throughout most of the set powers (Figure 2). This led to a setting of 6 W peak power (equals 1.5 W average power), in order to receive 1.2 W measured average power.

**Figure 4:**
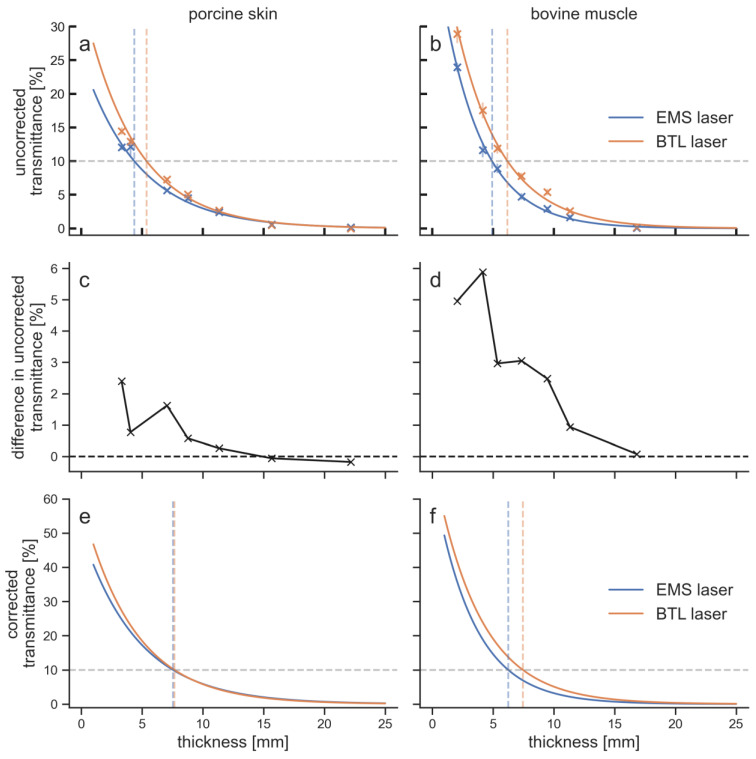
Penetration curves and differences in transmittance of the two LTDs (EMS laser, blue curves; BTL laser, orange curves) for the two tissues investigated: (**a**,**c**,**e**) porcine skin tissue; and (**b**,**d**,**f**) bovine muscle tissue. Transmittance was plotted against specimen thickness for the uncorrected data (**a**,**b**), the difference between the two LTDs in the uncorrected transmittance (**c**,**d**) and the corrected data (**e**,**f**). The 10% penetration depths are visualized in (**a**,**b**,**e**,**f**) by vertical dashed lines in the color of the LTD. The standard deviation of the measurements at each data point is given in (**a**,**b**) by vertical lines.

The BTL laser showed larger penetration depths than the EMS laser for both tissue types (Figure 4).

Especially for the upper 10 mm of tissue, the BTL laser achieved higher transmittances. The difference in transmittance between the EMS laser and the BTL laser decreased with increasing tissue thickness. The maximum difference in the uncorrected transmittances was 5.9% for bovine muscle tissue at 4.1 mm thickness and 2.4% for porcine skin tissue at 3.3 mm thickness. The differences were below 1% and therefore insignificant after 6.6 mm in porcine tissue and after 9.3 mm in bovine tissue. Each LTD penetrated the upper 5 mm of bovine tissue better than porcine tissue. The transmittance at 10 mm was for the BTL laser still larger in bovine tissue compared to porcine tissue, while for the EMS laser it was smaller in bovine tissue. For thicknesses of 15 mm and 20 mm, differences in transmittance were within the measurement error.

The sensor had a noise level of 2 mW, which can be translated to 0.2% of transmittance. The mean standard deviation of the measurements for the four penetration curves was between 0.3% and 0.7%. In addition, the error in the thickness measurements was approximately 0.2 mm. The overall measurement error was to some extent compensated through the curve fitting that resulted in the penetration curves; however, an error of 1% can safely be assumed for the transmittance values. The values of the penetration depth for different percentages as well as transmittances at different tissue thicknesses are given in Table 2.

**Figure 5:**
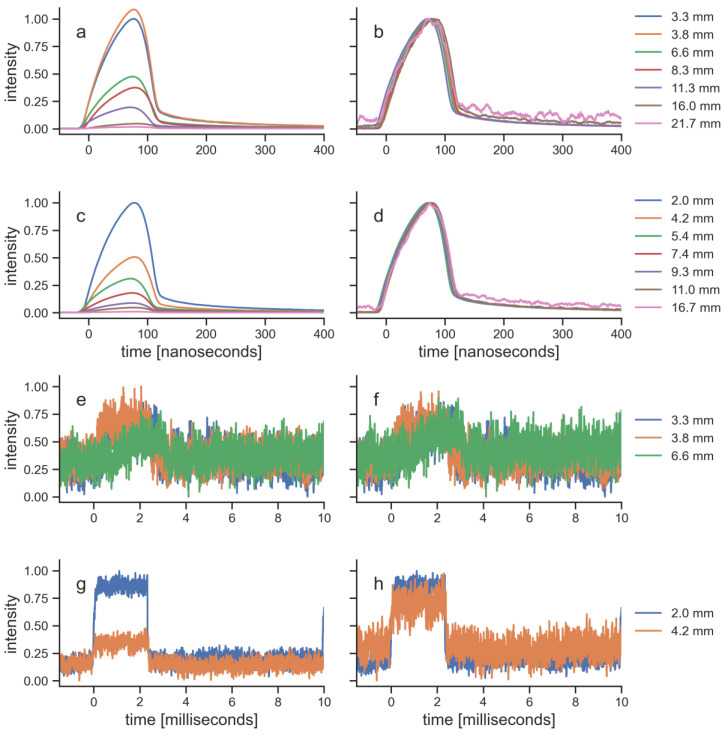
Recordings of laser pulses transmitted through tissue specimens: (**a**,**b**) EMS laser through porcine tissue; (**c**,**d**) EMS laser through bovine tissue; (**e**,**f**) BTL laser through porcine tissue; and (**g**,**h**) BTL laser through bovine tissue. The recordings were normalized in two ways: (**a**,**c**,**e**,**g**) normalized to the overall maximum of each laser therapy device and tissue; and (**b**,**d**,**f**,**h**) normalized to the individual maximum of each signal. All signals were smoothed using a moving average with a window length of 100 samples.

For each specimen, the pulses of the transmitted laser light were recorded as temporal profiles (Figure 5). Since the photodiode that was used for these measurements could not measure absolute values, the profiles were normalized in two ways (c.f. [8]): first to the maximum of the signal that was recorded with the thinnest specimen of each tissue and, secondly, to the maximum of each individual signal. The pulses of the EMS laser were able to transmit through all specimens, and were still recordable after transmission through the thickest specimen of both tissues. The pulse amplitude decreased with tissue thickness, while the pulse shape and pulse length stayed constant. In contrast, the pulses of the BTL laser were recordable only for the thinnest specimen of each tissue. The maximum thickness for a signal that was measured was similar for both tissues (porcine skin, 6.6 mm; bovine muscle, 5.4 mm). Thicker specimen did not allow recordings of the BTL laser pulses, because they were below the noise level of the photodiode. The signals from the five specimens that were recorded with the BTL laser also showed that the amplitude decreased with tissue thickness and the pulse shape stayed constant.

## 4. Discussion

This is the first study showing that 1064 nm laser light penetrated the upper 10 mm of biological tissue better than 905 nm laser light. For deeper tissue layers, the differences between the two wavelengths were insignificant. The same results were found for porcine skin and bovine muscle tissue. The hypothesis that 1064 nm light penetrates skin tissue but no other tissues better than 905 nm light was not confirmed. In contrast, the difference between the two wavelengths was larger in muscle tissue than in skin tissue.

The measurements described in the present study could only be performed with ex vivo tissue. A previous study showed that the penetration depths in porcine and bovine tissue were similar; only chicken tissue showed significantly larger penetration depths [8]. Since bovine and especially porcine tissues are more similar to human tissue than chicken tissue [16], these two tissue types were selected for the present study.

In the previous study [8], the uncorrected 10% penetration depths with the EMS laser were 4.2 mm for porcine skin (4.4 mm in the present study), and 5.0 mm and 5.1 mm for bovine muscle (4.9 mm in the present study). This high level of agreement between the two studies underlines the reproducibility of the used method.

Several studies used similar methods to investigate tissue penetration depths of NIR laser light into biological tissue [9,19,20,21,22,23]. The penetration depths found in the present study are comparable to the results from other studies (c.f. [8]). However, the only study that performed such measurements with 1064 nm laser light (or, in general, light with a wavelength greater than 1000 nm) used a custom-built sensor array to measure transmitted light [10]. The present study is the first one to demonstrate the transmission of laser light with a wavelength of 1064 nm through different types of tissue and compare the results with those obtained for laser light with a smaller wavelength (905 nm). Furthermore, important parameters such as wavelength, beam size, pulse length and average and peak power are often highly variable in studies of the penetration depth in biological tissues. These parameters were kept the same in the present study for both tissues and LTDs whenever possible. The fundamental differences between the two LTDs that were investigated in the present study were the wavelength (BTL laser, 1064 nm; EMS laser, 905 nm), the peak power (BTL laser, 12 W; EMS laser, 300 W) and the pulse length (BTL laser, 2.5 ms; EMS laser, 100 ns). We attempted to keep the beam size the same for both LTDs. However, since the beam size of the EMS laser could not be adapted and the setting for the beam size of the BTL laser did not function, the beam of the BTL laser was slightly larger (BTL laser, 20.0 mm; EMS laser, 18.7 mm). The beam size has an effect on the penetration depth, especially for small beams [12]. However, the penetration depth does not increase further for laser beams with a size larger than 10 mm [12,24]. Therefore, the differences in beam size between the EMS laser and the BTL laser could be neglected.

According to a study that used a similar method as the present study, PW laser light penetrates deeper into biological tissue than CW laser light [25]. However, it was found that this result was caused by erroneous measurements [26]. Others have reported that a higher repetition rate led to an increased penetration of 880 nm laser light [21]. However, there are several limitations to this finding. First, the differences were within the measurement error for both tissues. Secondly, not only the repetition rate was different (500 Hz and 71.4 MHz), but also the average power (2.55 W and 216 mW) and the beam size (3 mm and 11 mm) [21]. As discussed above, the beam size below 10 mm has a strong influence on penetration depth, therefore the results may have been influenced by this parameter. In our previous study [8], the penetration depth was determined using two LTDs at the same wavelength, beam size and average power. As in the present study, the repetition rate and the pulse length were not the same. However, no significant differences in penetration depth were found [8]. Therefore, it can be assumed that the findings of the present study were also not influenced by the repetition rate and pulse length. This leads to the conclusion that the difference in penetration depth is most likely due to the difference in wavelength.

The measured penetration curves were analyzed to compute penetration depths using the raw data and corrected data following the correction method from [8], which helps address reflection losses. Such correction methods can be used for small absorber and scatterer concentrations [27], and similar computations were performed in other, similar studies [8,10,22].

The absorption of light in generic tissue strongly depends on the wavelength. The main absorbing molecules within biological tissue are water, lipids, hemoglobin and melanin [7]. While the absorption coefficient of lipids is approximately equal at 905 nm and 1064 nm [7,14], the absorption coefficients of hemoglobin and melanin decrease with an increasing wavelength [7]. In contrast, the absorption of water is approximately two times larger at 1064 nm than at 905 nm [15], which at least partly compensates the effects of melanin and hemoglobin. Besides absorption, the scattering of light within the tissue also decreases the amount of light that can transmit to a target structure. Scattering is difficult to quantify since it depends on the highly variable fibrous character of a tissue [13]. In general, scattering is decreasing with longer wavelengths [7]. Therefore, the higher penetration depth of 1064 nm light compared to 905 nm light seen in the present study may be due to the lower absorption of hemoglobin and melanin and/or due to less scattering.

Since melanin has a lower absorption coefficient at larger wavelengths, and since melanin is only found in the epidermis, it has been hypothesized that 1064 nm light penetrates skin tissue better than light with a shorter wavelength [11]. It has been shown that the tissue penetration depth varies with skin color and therefore melanin content [16,28]. A more recent study [23] found a dependency of transmission through skin tissue with skin color only for 660 nm light, but not for 840 nm. The authors explained the results with the lower absorption of melanin at 840 nm [23]. Therefore, for the present study, skin and muscle tissues were compared using both 1064 nm and 905 nm light. In both tissues, the 1064 nm light similarly penetrated deeper than the 905 nm light. The different penetration depths of the two wavelengths can therefore not be explained by the absorption in melanin. However, the porcine skin tissue that was used for the present study was of a very light color, i.e., the melanin content in the epidermis was low. The same measurements should be performed using skin tissues with different melanin content in order to better understand this aspect. Regarding hemoglobin, one of the main limitations of the present study is that the investigated tissue contained deoxygenated hemoglobin compared to real tissue that would contain more oxygenated hemoglobin, since the absorption spectra for hemoglobin depends on the oxygen status [7]. However, all similar studies are limited by this since only ex vivo tissue with deoxygenated hemoglobin can be analyzed using common methods.

The penetration curves in the present study are based on measurements of the average power. However, the two LTDs emitted PW laser light. The way of pulsing of the LTDs was fundamentally different in terms of pulse shapes, pulse lengths, repetition rates and peak power. While the BTL laser emitted pulses with a length of 2.5 ms, a repetition rate of 100 Hz and a peak power of 12 W, the EMS laser pulses were 100 ns long with a repetition rate of 40 kHz and a peak power of 300 W. The pulse shapes and pulse lengths stayed constant as the light traveled through tissue (Figure 5), confirming the previous findings [8]. This implies that where the light from the EMS laser is reduced to 1%, the peak power is still 3 W, while the peak power of light from the BTL laser at 1% is reduced to 0.12 W. Of note, the important parameter at the target structure is the incoming power density, which further depends on the penetration depth since both LTDs had divergent laser beams. In general, using PW laser light is preferred over CW laser light as it reduces tissue heating of the surface layers and therefore allows higher peak power [17]. This effect is enhanced for reduced pulse lengths. Additionally, it has been hypothesized that the pulse length should be in the correct range for a local thermal activation of mitochondria and the rough endoplasmic reticulum, which is thought to be on the order of hundreds of nanoseconds [29]. These effects would therefore not be possible with the pulse length of the BTL laser, which is more than four orders of magnitude larger. Effects on the mitochondrial respiratory chain are often considered to be part of the underlying mechanisms in laser therapy, since the enzyme cytochrome c oxidase, which plays a role in the mitochondrial respiratory chain, is known to absorb red light [4].

Besides the activation of intracellular structures such as mitochondria, there are several other mechanisms of action of NIR laser light in laser therapy. However, despite many studies that addressed this question [30], the exact molecular and cellular mechanisms of action have remained poorly understood [2,4]. One of the main reasons for this is that many studies investigating these mechanisms focused on experiments in vitro, but there is a lack of corresponding studies in vivo and particularly clinical trials. For the treatment of musculoskeletal diseases with NIR laser light, three key mechanisms have been found so far in studies in vivo (c.f. [5]).

First, treatment with laser light may lead to an analgesic effect. Specifically, it was shown that laser light can influence the firing rate of nociceptors [31]. The authors of the latter study recorded the activity of heat nociceptors in the tongue of anesthetized cats that were heated by a thermostatically controlled thermal probe. A pretreatment with PW laser light (wavelength, 904 nm; peak power, 2 W; pulse length, 200 ns; repetition rate, 3040 Hz) substantially decreased the nociceptors’ firing rates [31]. An analgesic effect was also observed in the laser light treatment of rats that had a surgical injury to the articular disc of the temporomandibular joint [32]. In the latter study, treatment with PW laser light (wavelength, 904 nm; peak power, 70 mW; pulse length, 60 ns; repetition rate, 9500 Hz) caused a total decrease in pain sensitivity, together with a significant decrease in substance P, vanilloid transient potential receptor 1 (TRPV-1) and calcitonin gene-related peptide (CGRP) compared to the control animals [32]. Similar results were found for rats after a chronic constriction injury that underwent treatment with the same laser parameters [33]. Substance P and CGRP are inflammatory mediators from afferent nerve fibers that play a role in neurogenic inflammation [34,35,36]. TRPV-1 is known as a nonselective cation channel for radial heat and capsaicin [37]. In another study, mice in which the gene encoding substance P was deleted did not show an analgesic effect of NIR laser treatment (wavelength, 685 nm; laser mode (CW or PW) not mentioned; output, 30 mW) compared to wild-type mice [38]. In addition, the TRPV-1 antagonist capsazepine inhibited the analgesic effect of the laser treatment. To our knowledge, similar studies using a 1064 nm NIR laser have not been reported.

Second, NIR laser treatment has an effect on prostaglandin E2 (PGE2) concentrations in peritendinous tissue [39]. The authors of the latter study investigated the PGE2 concentrations in the peritendinous tissue parallel to the Achilles tendon of human patients suffering from bilateral Achilles tendinitis. One Achilles tendon of each patient received laser pretreatment (wavelength, 904 nm; peak power, 10 W; pulse length, 200 ns; repetition rate, 5000 Hz). Sham treatment was performed on the other Achilles tendon of each patient. Then, the patients had to run on a treadmill, and the PGE2 concentrations in the peritendinous tissue of both Achilles tendons were evaluated using microdialysis. It was found that the laser pretreatment resulted in lower PGE2 concentrations even during exercise, which increased the PGE2 concentrations on the sham-treated side [39]. PGE2 plays a central role in inflammation and pain perception via inflammatory nociception, in which PGE2 sustains inflammation and pain [40,41]. Furthermore, PGE2 can pre-sensitize the TRPV-1 channel and, thus, may aggravate neurogenic inflammation and pain recognized by the unmyelinated C nerve fibers [42]. Again, to our knowledge, similar studies using a 1064 nm NIR laser were not reported.

Third, it was shown that early laser treatment after structural muscle trauma can reduce the formation of scar tissue [43]. After structural gastrocnemius muscle injury in a rat model, early treatment with a CW laser (wavelength, 904 nm; PW laser mode; pulse length and peak power not mentioned; average power, 45 mW) reduced the inflammatory response [43]. Additionally, the laser treatment blocked the activation of the transcription factor nuclear factor kappa B (NF-κB) and the effects of reactive oxygen species release [43]. NF-κB is active in many inflammatory diseases, including atherosclerosis and others [44], and anti-inflammatory activity has been linked to a suppression of NF-κB activation [45]. Of note, the release of PGE2 may be promoted by an increase in NF-κB translocation [46]. Therefore, the reduction of PGE2 concentrations after laser treatment (that was shown in [39]) may be related to the effect of laser treatment on NF-κB [30,46]. Again, to our knowledge, similar studies using a 1064 nm NIR laser were not reported.

The described mechanisms of action were all based on studies that used PW laser light with a wavelength of 904 nm or smaller. There is little information about the cellular and molecular effects of light with a wavelength greater than 1000 nm [12]. Without knowledge of whether 1064 nm laser light has the same or similar anti-inflammatory effects or effects on nociceptors as 904/905 nm laser light, the higher transmittance of 1064 nm than 905 nm laser light within the first 10 mm of biological tissue may be clinically irrelevant.

Finally, the performance of the BTL laser in the beam characterization measurements needs to be addressed. Comparing light emission to the settings at the device revealed several issues with the BTL laser. First, the emitted power was consistently below the set power by 5–20% (Figure 2). In comparison, the maximum deviation of the measured to set power of the EMS laser as determined in a previous study was 3.3% [18]. Large differences between the set and emitted power have been observed with a different LTD before [18]. Since the deviations of the BTL laser were relatively consistent for all tested modes, it can be hypothesized that this originated from deteriorations such as damages to the fiber optical cable or an unclear lens (the tested device was new). These deteriorations could also have led to the high noise levels of the spatial intensity distribution of the BTL laser (Figure 3c). The spatial intensity distribution of the EMS laser was a flat top distribution [8,18], in contrast to the Gaussian distribution of the BTL laser. The main issue with the BTL laser, however, was that the setting to change the beam size was fully dysfunctional (Figure 3d). Whether the function to change the beam size of the BTL laser was dysfunctional only in the unit used in the present study or whether this is a general problem of this LTD was not further investigated. Nevertheless, this reaffirms the need for better standardization and verification of LTDs [18,47,48].

## 5. Conclusions

The analysis of light transmittances in biological tissue using two laser therapy devices revealed that light with a 1064 nm wavelength penetrates the upper 10 mm of tissue better than light with a 905 nm wavelength. However, the pulsing mode and peak power of the light seems to be of greater importance than the wavelength when it comes to efficiently transporting light into deep layers of tissue. The mechanisms of action of NIR light at these tissue layers remain poorly understood. However, the in vivo evidence that exists has been acquired using 904 nm laser light, and whether the same mechanisms apply for 1064 nm light needs to be addressed in future studies. Overall, laser therapy is an emerging modality in the treatment of musculoskeletal diseases. Since laser therapy offers several advantages such as being non-invasive, free of many side effects reported for drugs and treatments using other medical devices, and free of drugs, it should be further investigated, especially in experiments in vivo and particularly clinical trials. Further investigations should be performed with special attention regarding wavelength, peak power and pulse length of the used LTD.

## Figures and Tables

**Table 1:** Details of the two laser therapy devices that were the subject of the present study. Values for average power ($P_{average}$), peak power ($P_{peak})$, pulse length and repetition rate were taken from the user manuals of the manufacturers.

Laser Therapy Device	EMS Laser	BTL Laser
Wavelength [nm]	905	1064
Modes	PW	CW/PW/SP
max. $P_{average}$ [W]	1.2	12
$P_{peak}$ [W]	300	12
Pulse length	100 ns	2.5–250 ms
Repetition rates [Hz]	5000–40,000	1–100

Abbreviations: nm, nanometer; W, Watt; Hz, Hertz; PW, pulsed wave; CW, continuous wave; SP, single pulse.

**Table 2:** Penetration depths and transmittances for the two laser therapy devices and both tissues. Values were computed from both uncorrected and corrected penetration curves.

Laser	Tissue	Penetration Depth [mm] for				Transmittance [%] at			
		15%	10%	5%	1%	5 mm	10 mm	15 mm	20 mm
**uncorrected**									
BTL laser	porcine skin	3.6	5.4	8.4	15.3	10.9	3.4	1.1	0.3
	bovine muscle	4.7	6.2	8.8	14.9	13.7	3.7	1.0	0.3
EMS laser	porcine skin	2.5	4.4	7.6	15.1	8.7	3.0	1.0	0.3
	bovine muscle	3.6	4.9	7.2	12.5	9.8	2.1	0.5	0.1
**corrected**									
BTL laser	porcine skin	5.9	7.7	10.7	17.6	18.5	5.8	1.8	0.6
	bovine muscle	5.9	7.5	10.1	16.2	19.1	5.1	1.4	0.4
EMS laser	porcine skin	5.7	7.5	10.8	18.3	17.3	5.9	2.0	0.7
	bovine muscle	4.9	6.3	8.5	13.8	14.6	3.2	0.7	0.2

## Data Availability

The data used during the present study are available from the corresponding author upon reasonable request.
